# Cognitive-behavioral family therapy as psychoeducation for adolescents with high-functioning autism spectrum disorders: Aware and Care for my Autistic Traits (ACAT) program study protocol for a pragmatic multisite randomized controlled trial

**DOI:** 10.1186/s13063-020-04750-z

**Published:** 2020-09-29

**Authors:** Fumiyo Oshima, Mandy William, Noriko Takahashi, Aki Tsuchiyagaito, Hitoshi Kuwabara, Akihiro Shiina, Mikuko Seto, Minako Hongo, Yui Iwama, Yoshiyuki Hirano, Chihiro Sutoh, Kayoko Taguchi, Tokiko Yoshida, Yohei Kawasaki, Yoshihito Ozawa, Jiro Masuya, Noriyuki Sato, Shizuka Nakamura, Masaru Kuno, Jumpei Takahashi, Toshiyuki Ohtani, Daisuke Matsuzawa, Naoko Inada, Miho Kuroda, Mika Ando, Arinobu Hori, Akiko Nakagawa, Eiji Shimizu

**Affiliations:** 1 Research Center for Child Mental Development, Chiba University, 1-8-1 Inohana Chuouku, Chiba, 260-8670 Japan; 2 Research Department of Clinical, Educational & Health Psychology, University College London, London, UK; 3 Fukushima University Child Mental Health-Care Center, Fukushima, Japan; 4 Laureate Instituto for Brain Research, Tulsa, OK USA; 5 Department of Psychiatry, Hamamatsu University School of Medicine, Shizuoka, Japan; 6 Chiba University Center for Forensic Mental Health, Chiba, Japan; 7 Biostatistics Section, Clinical Research Center, Chiba University Hospital, Chiba, Japan; 8 Department of Psychiatry, Tokyo Medical University Ibaraki Medical Center, Ibaraki, Japan; 9 Department of Psychology, Faculty of Liberal Arts, Teikyo University, Tokyo, Japan; 10 Department of Human Care, Nagoya University of Arts and Sciences, Nagoya, Japan; 11 Department of Psychiatry, Hibarigaoka Hospital, Fukushima, Japan; 12 Hori Mental Clinic, Fukushima, Japan

**Keywords:** High-functioning autism spectrum disorder, Randomized controlled trial, Cognitive-behavioral family therapy, Psychoeducation

## Abstract

**Background:**

One aim of an autism spectrum disorder (ASD) diagnosis is to obtain special support for the disorder, though this does not guarantee practical support. We developed a psychoeducational program using cognitive-behavioral therapy (CBT) and Aware and Care for my Autistic Traits (ACAT) for Japanese adolescents with high-functioning ASD and their parents.

**Methods:**

This multisite study is a randomized controlled trial. In total, 24 participants will be assigned to the ACAT group and 24 to the treatment-as-usual (TAU) group. The ACAT group will receive a weekly 100-min session for 6 weeks, regular medical care, and one follow-up session. In this ongoing clinical trial, we will compare the scores of the measures recorded in the pre- and post-intervention stages between the ACAT and TAU groups. A total of 41 patients out of a target of 48 have participated in the trial to date. The primary outcome measure is the Autism Knowledge Questionnaire. Secondary outcome measures include Barriers to Access to Care Evaluation 3rd Edition, the Strengths and Difficulties Questionnaire, the Vineland Adaptive Behavior Scales second edition, the Parenting Resilience Elements Questionnaire, the General Health Questionnaire 12, and the Depression Self-Rating Scale for Children assessments, as well as an electroencephalographic recording.

**Discussion:**

It is expected that participants in the ACAT group will significantly increase their self-understanding and awareness of ASD symptoms compared to those in the TAU group. Additionally, the ACAT group is expected to exhibit improved social adaptation and mental health if children and parents are able to better understand the ASD characteristics through sessions. This intervention will contribute to the establishment of an effective evidence-based treatment strategy for adolescents with ASD.

**Trial registration:**

UMIN Register 000029851. Registered on January 06, 2018

## Background

Autism spectrum disorder (ASD) is a pervasive neurodevelopmental disorder characterized by impairments in social communication and restricted, repetitive patterns of behavior, interests, or activities [1]. ASD is a relatively common neurodevelopmental condition that affects 1–1.5% of the population [2, 3]. Individuals with high-functioning ASD (HF-ASD) have normal-range IQs and extensive verbal abilities and usually attend regular schools. Nevertheless, their difficulties are potentially serious and can include impaired social relations, obsessions, and uneven levels of intellectual and cognitive functioning [4]. When patients with HF-ASD become independent and increase social interactions as they enter adolescence, they become aware of their differences to their surroundings, which tends to lower their self-efficacy and self-esteem and increase feelings of depression and anxiety [5–7].

An extensive treatment has recently been made available to support the central characteristics of ASD early in life, as evidenced by the Early Start Denver Model [8] and parent-mediated communication-focused treatment in children with autism (PACT) [9]. These treatment programs are not available for people with autism post-early childhood. However, individuals with autism who are diagnosed later may experience benefits in social functioning by gaining support, such as social skills training [10, 11] and/or reasonable accommodation [12].

In childhood and adolescence, individuals without knowledge of focal features and those with HF-ASD can easily experience self-stigma such as feeling “abnormal” or “inferior” [13]. As stigma acts as a barrier to help-seeking behavior [14], patients may experience reduced opportunities to obtain support alongside increased feelings of experiencing difficulties [15].

Conversely, the provision of psychoeducation on ASD has been reported to decrease adverse feelings toward children with the disorder. Gaining knowledge on ASD is also extremely important to the parents. Farrugia reported that parents who gained medical knowledge about ASD experienced decreased stigma toward children with ASD [16]. In addition, Gordon et al. [17] demonstrated that participation in a brief parent-child program of psychoeducation, the PsychoEducational Groups for Autism Spectrum Understanding and Support (PEGASUS), which sought to promote self-understanding and acceptance of HF-ASD, increased self-awareness in both parents and children. The PEGASUS is a psychoeducation group for young individuals with HF-ASD and their parents that promotes awareness of autistic traits. However, it cannot help them cope with behavioral problems in multiple domains. Therefore, we conjectured that cognitive-behavioral family therapy (family CBT) may help HF-ASD children/adolescents and their families to understand the central characteristics of ASD and cope with the cognitive and behavioral problems caused by autistic traits. The CBT treatment seeks to produce cognitive alterations in various ways to endure emotional and behavioral changes [18]. Reports have suggested that the application of CBT for ASD represents a major advance in the treatment of co-occurring anxiety and depression in HF-ASD patients [6, 19, 20]. There is a small body of literature indicating that CBT is effective for managing problems faced by young individuals with ASD, such as anger, anxiety, social interaction, and emotional understanding [21]. For children with ASD, modifying the standard CBT protocol is necessary to induce general and permanent changes [22]. In this regard, we have included family participation because the parents need to understand the distinct features of their children’s AD to understand the considerations required when caring for them.

For this study, the authors developed a new intervention for HF-ASD children/adolescents termed the Aware and Care for my Autistic Traits (ACAT) program. This program includes psychoeducation and family CBT for HF-ASD children/adolescents and their parents/guardians. The details of this program are explained in the “Methods/design” section.

Since the ACAT program is based on psychoeducation that augments family CBT, the primary outcome is the increased awareness of ASD measured by the child/youth version of the Autism Knowledge Quiz (AKQ) [17]. In addition, a social adaptation scale and a stigma scale are collected as secondary outcomes to monitor the relative improvement of these two factors. Moreover, investigating the relationship between behavioral and neural changes after the intervention can provide strong evidence that the intervention has an impact on behavioral and neural substrates. Therefore, we will record resting-state electroencephalograms (EEG) as a neural outcome in this study for exploratory purposes. Although the causes and biomarkers of ASD remain unclear, evidence suggests that ASD is a neurobiological disorder underscored by abnormal brain activity related to the observed social deficits [23]. EEG, which primarily measures neurophysiological changes related to postsynaptic activity over the cortex, can capture and characterize abnormal brain activity. For example, individuals with ASD showed increased short-range connections in the lateral-frontal electrodes and decreased long-range connections in the fronto-occipital electrodes compared with healthy individuals [24]. Moreover, other studies have shown that altered EEG patterns are normalized in response to improved social behaviors after social training programs, which indicates that psychosocial interventions have the potential to alter brain activity as measured by EEG.

### Objectives

This study aims to examine the efficacy of the newly developed psychoeducation program for parents and individuals with HF-ASD, or ACAT, by comparing with the treatment-as-usual (TAU) group. Individuals and parents who are assigned to the ACAT group will keep their current treatments, which the ACAT program will augment. We hypothesize that the ACAT group will show greater children and parents’/guardians’ understanding and awareness of their autistic attributes than the TAU group. Randomized controlled trials will be conducted at multiple sites to investigate the relative efficacy of ACAT compared to TAU.

Our goal is to examine the relative efficacy of the combination of psychoeducation and family CBT for both child/adolescent patients with HF-ASD and their parents/guardians after diagnosis. This study will help to establish a new evidence-based psychoeducational treatment strategy for child/adolescent patients with HF-ASD and their parents/guardians. It will enable more treatment options as an after-diagnosis service and improve the dissemination of psychoeducation and CBT for HF-ASD.

## Methods/design

### Study design

This study is designed as a prospective randomized open-label endpoint trial with two parallel intervention groups consisting of a 6-week treatment of treatment-as-usual (TAU group) alone and an ACAT combined with TAU (ACAT group). It is randomized, multisite, stratified, single-blinded, and is conducted through a parallel between-group design.

Qualified individuals who consent to this trial are randomly assigned to a TAU sustained group or a COMB group that combines psychoeducation and family CBT. The study is planned in accordance with the Standard and Protocol Items: Recommendations for Interventional Trials [25] and will be analyzed and reported in line with the Consolidated Standards of Reporting Trials Recommendations [26].

### Participants

The eligible participants in the current study are individuals diagnosed with ASD and their parents or guardians. Before the final inclusion, both written and verbal informed consent is obtained from all participants in the study.

#### Inclusion criteria

The participants in this study comprise patients and their parents/guardians who meet the following criteria during the screening process:

*Individual child/adolescent with ASD:*

1. Meet the cutoff value for ASD measured using the Autism Diagnostic Observation Schedule [27] or Autism Diagnostic Interview-Revised [28]
2. Have a verbal IQ of 90 or above as measured by the Wechsler Intelligence Scale for Children [29]
3. Have a social difficulty assessment of “some need” or higher measured by the Strengths and Difficulties Questionnaires [30]
4. Aged between 10 and 17 years at the time of consent, with one of the parents being eligible to participate in psychoeducation and CBT (if assigned to COMB)
5. Diagnosed with ASD by a primary doctor or other doctors or rehabilitation center for children
6. Received outpatient psychiatric care
7. Able to understand CBT and is mentally and physically able to sustain therapy for at least 2 months

*Parents/guardians of ASD individuals:*

1. Living with the ASD individual as a parent or a grandparent and acts as a guardian
2. Able to participate in CBT once a week with the ASD individual

#### Exclusion criteria

*Individual child/adolescent with ASD:*

1. Current active suicidal intent
2. Active repetitive antisocial behavior
3. Active severe degenerative physical disorder that may disrupt CBT

*Parents/guardians of ASD individuals:*

1. Must not meet the diagnostic criteria for psychiatric disorders measured by the Mini-International Neuropsychiatric Interview [31] to be eligible for participation in the study
2. Unable to accompany the patient to the sessions

### Procedures

Beginning in March 2018, recruitment, treatment, and data collection are being conducted in two cities in Japan (Chiba and Fukushima). The last follow-up treatment will be completed in August 2020. Children and adolescent outpatients (10–17 years of age) diagnosed with ASD and their parents and guardians are informed about the intervention in waiting rooms at Chiba University Hospital and a local psychiatry clinic. For those who show interest, more information is provided by the recruitment staff.

**Fig. 1:**
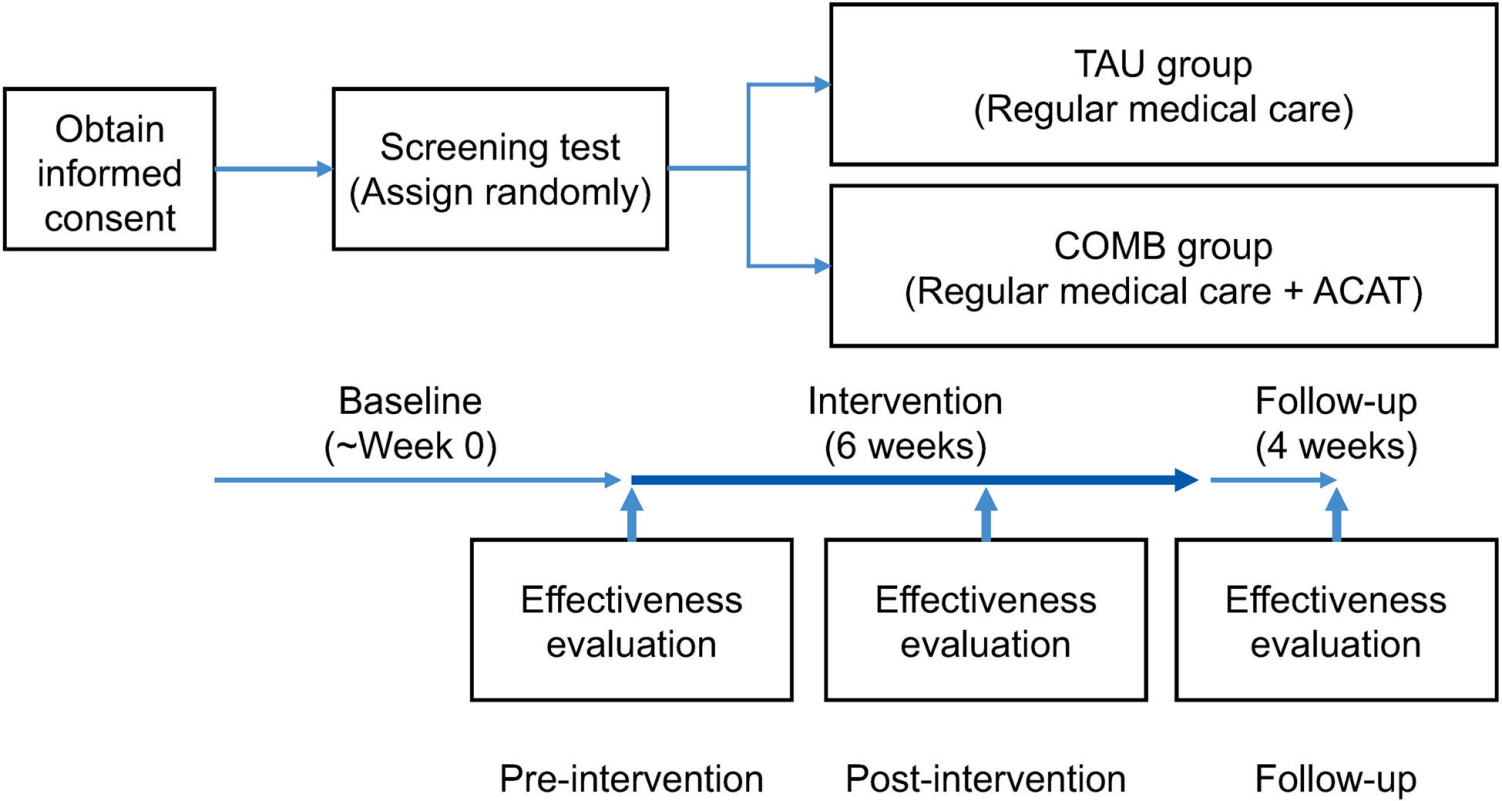
CONSORT flow chart of the clinical trial

The flow chart of this clinical trial is shown in Fig. 1. The trial will be conducted over 6 weeks of interventions and 4 weeks of follow-up. To evaluate treatment efficacy and safety, outcome measure assessments will be administered before and after the interventions. The participants assigned to COMB will continue their usual treatment with their primary doctors and also participate in six sessions of the ACAT program (a 100-min psychoeducation and family CBT program developed by the authors) every week (the ACAT program is conducted for a duration of 6 weeks). Participants taking psychotropic drugs regularly as medical treatment will be requested to remain on stable dosage during the interventions; however, the dosage may change if their primary or prescribing physicians consider it medically necessary. Any change in the dosage will be recorded in the list of daily doses of psychoactive drugs. A follow-up interview will be conducted 4 weeks after the completion of the interventions (10th week). Interviews and symptom assessments will be undertaken at each facility during week 0 (pre-intervention), week 6 (post-intervention), and week 10 (follow-up). The participants assigned to the TAU group will be required to continue their usual treatment with their primary doctors. Furthermore, a combination of the following treatments is prohibited with their usual treatment: psychotherapy that conforms to CBT or physiological therapies, including electroconvulsive therapy and magnetic stimulation therapy. The interviews and symptom assessments at pre-intervention, post-intervention, and follow-up will be conducted for TAU at each facility in the same manner as that for COMB. The restrictions for psychotropic drugs are the same as those for the COMB group. All sessions and psychological examinations are free for the participant. Both child/adolescent patients and their parents/guardians will receive 10,000 yen (about 100 USD) after completion of the last measurement. Any harm that occurs will be reported after the study period, whether or not it could have been related to the study treatment. Patients will be observed for up to 4 weeks (after discontinuation). To date, 41 patients in total have been randomized.

### Randomization and blinding

Enrollment and randomization are based on the central registration system of the Clinical Research Data Center at Chiba University Hospital, Chiba, Japan. Two allocation factors are used for stratification: the degree of knowledge (high or low) regarding the characteristics of ASD according to the primary outcome measure, the Autism Knowledge Quiz-Child (AKQ-C), and gender (male or female). At the end of the baseline assessment, eligible participants will be randomly assigned to either the TAU arm or ACAT plus TAU arm at a ratio of 1:1, with assignments made using the minimization method, ensuring a balance across baseline AKQ scores and gender. Each participant will then be assigned to one of the two treatments. Participants will not be blinded to the group to which they are assigned before consenting to participate in the study. However, trained testers, who will assess all outcome measures except the self-assessment scale, will be blinded to the group.

### Data monitoring

An Independent Data Monitoring Committee has been established for this study. It will meet every 3 months to review a report created by the researchers to monitor the progress of recruitment and data collection (e.g., completion rates for each follow-up).

### Ensuring the quality of therapists’ skills and sessions

Psychiatrists and clinical psychologists, licensed for at least 3 years and involved in clinical work with ASD individuals, have been engaged as cognitive-behavioral therapists for this study. The therapists for ACAT received at least 6 h of initial training on the intervention, read the treatment manual, listened to the audiotape set of the model therapist performing the treatment, and are supervised weekly by the clinical supervisor (the first author of this thesis who developed the ACAT protocol). All sessions will be recorded and evaluated by researchers unrelated to the development or operation of the ACAT program using the Cognitive Therapy Scale [32].

### ACAT program details

This study follows the ACAT protocol shown in Table 1. We started to develop a psychological education program for ASD, referring to programs for ASD outside of Japan that can be adopted as the standard in Japan.

**Table 1:** Agenda and purpose of each ACAT session

CBT sessions	Session content	Session goals
Pre-session	Learn about your psychological assessment for ASD	The participants will learn about their personal characteristics and autistic traits through the results of assessment tests.
1st CBT	Learn about the characteristics of your autistic traits	The participants will understand the differences between autistic attributes and typical developmental features.
2nd CBT	Cognitive changes: learn not only about the “weakness” but also “strengths” of your autistic traits	The participants will discover the “strengths” of ASD, setting aside the “weaknesses” and turning negative perspectives into rational neutral understanding about themselves.
3rd CBT	Cognitive changes: create “funny nicknames” for your autistic traits	The participants will learn to externalize their autistic characteristics, facilitate self-monitoring skills, and develop metacognition.
4th CBT	Behavioral changes: create a problem-solving plan for your autistic traits	The participants will learn how they can change and adjust their problematic behaviors to cope with their autistic traits.
5th CBT	Behavioral changes: plan a functional way to solve easy problems that are caused by your autistic traits with your parents	The participants will make problem-solving plans for their own autistic traits and will also consider how to cope with problems when they execute their plans. They will start with easier problems related to their ASD traits. They will conduct these plans as a homework assignment.
6th CBT	Behavioral changes: plan a functional way to solve more complicated problems that are caused by your autistic traits with your parents	The participants will make problem-solving plans for their own autistic traits and will also consider how to manage more complicated problems when they execute their plans. They will conduct these plans as a homework assignment (this homework assignment will be checked at a follow-up session).
CBT follow-up	Check how well you are living with your autistic traits	The participants will review all skills and techniques learned to date. They will check their understanding of their own autistic traits and discuss the progress made in therapy, areas of continued effort, and ongoing challenges.

The concept of the program is to help adolescents with ASD and their caregivers learn about ASD and improve their skills in coping with “ASD traits” using CBT. The mechanism of the expected effect is that it will increase and consequently eliminate social and psychological difficulties. The ACAT protocol was preliminarily utilized in two patients with ASD and their parents to test it in clinical situations and collect feedback, after which it was revised. The ACAT program is conducted over a 100-min session per week with a 10-min break, with six sessions in total. An individual with ASD and a parent/guardian attends the session together. An agenda is prepared for each session (Table 2). The workflow is described in Table 1. The first three sessions focus on participants learning about their own ASD characteristics and their ability to externalize these characteristics by creating funny nicknames, which facilitates self-monitoring skills and development of metacognition of their characteristics. From the fourth session onward, featured difficulties (symptoms) arising from their autistic traits are discussed. These procedures reasonably accommodate the individual abilities of each participant. Each session has a homework assignment that focuses on reviewing the discussed material. If a participant forgets to do a homework assignment or is absent or late to a session for any reason, a make-up session will be provided to fill in the gaps.

**Table 2:** Agenda for each session

Minutes (total 100)	Contents of each session
10	Check the condition and mood over the week (0–100 evaluation) based on the ACAT textbook
20	Check homework assignments
20	Read the ACAT textbook related to the day’s topic (about the day’s topic, see Table 1)
10	Break
30	Practice work (e.g., cognitive and behavioral intervention) related to the day’s topic based on the ACAT textbook
5	Set homework assignment that is related to the day’s topic
5	Ask the patient and parent for feedback about the session

### Schedule for screening tests

Table 3 describes the operational schedule for the screening test and outcome measures.

**Table 3:** Standard Protocol Items: Recommendations for Interventional Trials (SPIRIT) diagram

Time point**	Study period							
	Enrollment, ***May 2017***_***1***_	Allocation, March 2018	Post-allocation					Close-out, ***May 2020***
			***0w***	***6w***	***10w***	***t***_***4***_	etc.	
**Enrollment:**								
**Eligibility screen**	X							
**Informed consent**	X							
**Allocation**		X						
**Interventions:**								
***Combination (ACAT) group***			X	X	X			
***TAU group***			X	X	X			
**Assessments:**								
***Socioeconomic and demographic data***	X	X	X	X	X			
***Patients: AKQ-C, DSRS-C, BACEv3, EEG***	X	X	X	X	X			
***Parents: AKQ-P, SDQ, Vineland-2, Bace-v3, GHQ-12, EEG***	X	X	X	X	X			

*AKQ-C* Autism Knowledge Quiz-Child, *DSRS-C* Depression Self-Rating Scale for Children, *BACEv3* Barriers to Access to Care Evaluation scale version 3, *EEG* electroencephalogram, *AKQ-P* Autism Knowledge Quiz-Parents, *SDQ* Strengths and Difficulties Questionnaire, *GHQ-12* General Health Questionnaire

Screening tests will be conducted for participants and their parents after obtaining their informed consent. A physician or therapist will perform the screening tests as shown in Table 3. Patients who qualify according to the selection criteria and do not meet the exclusion criteria will be selected. As inspection items, we will ascertain the backgrounds and names of participants and dosage of psychotropic drugs administered to them along with their usage period. We will conduct the Autism Diagnostic Observation Schedule Second Edition (ADOS-2) [27] and Autism Diagnostic Interview-Revised (ADI-R) [28] to assess ASD and the ADHD Rating Scale-IV (ADHD-RS) [33] to assess hyperactivity and impulsivity accompanying their ASD. We will conduct the Japanese version of the Sensory Profile [34] to assess dysesthesia and administer the Strengths and Difficulties Questionnaire (SDQ) [30] to assess behavioral problems. We will also conduct the Wechsler Adult Intelligence Scale-III (WAIS-III) or Wechsler Intelligence Scale for Children, Fourth Edition (WISC-IV) [35] according to the patient’s age to assess their intelligence level. Further, the Mini International Neuropsychiatric Interview (M.I.N.I.) [31] will be used to screen the parents of participating patients for the presence of diagnostic criteria, including panic disorder, agoraphobia, general anxiety, and depression.

### Outcome measures

#### Primary outcomes

##### Autism Knowledge Quiz-Child (AKQ-C)

AKQ-C [17] has two sections, namely, a five-item structured interview on “awareness of autistic characteristics” and a 15-item knowledge quiz. The main evaluation items of this study include variations in the “awareness of autistic characteristics” measured in section 1 of the AKQ-C, which will be administered during the pre-intervention, post-intervention, and follow-up period.

#### Secondary outcomes

##### Depression Self-Rating Scale for Children (DSRS-C)

DSRS-C [36] is a self-assessment scale containing 18 items. It assesses the level of depression among children by enquiring about their feelings over a week; each item is marked on a three-point scale: “always,” “sometimes,” and “never.” This scale will be applied to children/adolescents at three different time points: pre-intervention, post-intervention, and follow-up.

##### Barriers to Access to Care Evaluation scale version 3 (BACE-3)

The BACE [37] is a 30-item questionnaire designed to assess the barriers to mental health care for people with mental health problems. It includes barriers related and unrelated to stigma and discrimination. This scale will be applied to both children/adolescents and parents/guardians at three different time points: pre-intervention, post-intervention, and follow-up.

##### Strengths and Difficulties Questionnaire (SDQ)

SDQ [30] is a short screening instrument that addresses the positive and negative behavioral attributes of infants, children, and adolescents. It includes 25 items, and each item can be marked “not true,” “somewhat true,” or “certainly true.” The scale will be applied to parents/guardians who are requested to score their children/adolescents at three different time points: pre-intervention, post-intervention, and follow-up.

##### Vineland Adaptive Behavior Scales second edition (Vineland-2)

Vineland-2 [38] is a semi-structured interview for parents to capture the developmental norm of adaptive behavior in individuals aged 0–92 years. Interviews will be conducted with parents/guardians about their children at three different time points: pre-intervention, post-intervention, and follow-up.

##### Autism Knowledge Quiz-Parents (AKQ-P) (for parents/guardians)

AKQ-P [17] is the parent version of AKQ-C. We will apply this scale to parents/guardians at three different time points: pre-intervention, post-intervention, and follow-up.

##### The 12-item General Health Questionnaire (GHQ12) (for parents/guardians)

GHQ [39] is a measure of current mental health. The questionnaire was originally developed as a 60-item instrument; however, a range of shortened versions of the questionnaire such as GHQ-30, GHQ-28, GHQ-20, and GHQ-12 are available. The scale tests if respondents recently experienced a particular symptom or behavior. Each item is rated on a 4-point scale (less than usual, no more than usual, rather more than usual, or much more than usual); for GHQ-12, it gives a total score of 36 or 12 based on the selected scoring methods. We will apply this scale to parents/guardians at three different time points: pre-intervention, post-intervention, and follow-up.

##### Parenting Resilience Elements Questionnaire (PREQ) (for parents/guardians)

PREQ [40] measures the degree to which mothers possess elements that aid in adapting to challenges and difficulties related to children with developmental disorders. It comprises 29 items and three factors, namely, “knowledge of the child’s characteristics,” “perceived social supports,” and “positive perceptions of parenting.” Each item is scored on a 7-point Likert scale from 1 (strongly disagree) to 7 (strongly agree). We will apply this scale to parents/guardians at three different time points: pre-intervention, post-intervention, and follow-up.

##### Electroencephalogram (EEG)

An EEG analysis can be used to detect the disrupted functions of large-scale cortical activity in ASD patients and to provide objective indices to evaluate the effectiveness of this program [41]. Therefore, resting-state EEG will be recorded as an objective physiological index alongside the questionnaire. The EEG scale will be applied to both children/adolescents and parents/guardians at three different time points: pre-intervention, post-intervention, and follow-up.

### Other measurements

Before and after treatment, parents are requested to report their children’s current medication, dose of medication, and outpatient (hospital) care.

### Sample size

The sample size was calculated using the G*power software version 3.1.9.2 with a power of 0.80 and *α* = 0.05 (two-tailed). In a previous study [17], the change in AKQ’s “ASD awareness” was 2.26, and the effect size was 0.92. Considering a drop out ratio of 20%, the ideal sample size was estimated as 48.

### Statistical methods

#### Statistical analysis plan

We are currently collecting data from 41 of 48 child/adolescent ASD patients and their parents/guardians. Treatments will end during the spring of 2020, and analysis of the data will commence in the summer of 2020. The purpose of this study is to examine the relative efficacy of ACAT for child/adolescent ASD patients and their parents/guardians, in terms of ASD awareness scores, compared to the TAU group. Therefore, the primary analysis will evaluate the differences in score variations (i.e., the ACAT and TAU groups’ mean variations) for ASD awareness from week 0 (baseline) to week 6. Relative efficacy will be indicated when the point estimate of mean variation difference has a positive value and when the value of the ACAT group statistically exceeds that of the TAU group. The examination of scores will be based on an analysis of covariance (ANCOVA) for interval estimation of mean variation differences and calculation of the P value using allocation factors as adjustment factors (gender and AKQ-C score). The secondary evaluation of efficacy items will supplement the main results of the analysis, and redundancy will not be adjusted. The level of significance for the hypothesis test is 5% on two sides, with a two-sided confidence interval of 95%.

The results of the BACE-3, AKQ-P, SDQ, Vineland-2, DSRS-C, PREQ, and GHQ-12 (self-completed by parents) will be analyzed using the same techniques used for the assessment of the main evaluation items. In addition, EEG of individuals with HF-ASD as well as their parents/guardians will be recorded at pre-intervention, post-intervention, and follow-up to investigate neurological changes. EEG signals will be registered with a 19-channel box of Mitsar-EEG 201 (Mitsar Co. Ltd.). The 19-channel EEG cap will be fitted in accordance with a standard 10–20 system. EEG analysis will be conducted based on graph theory. The overall efficiency, clustering coefficients, connection between the left and right hemispheres, and network resilience will be calculated and compared between the ACAT and TAU groups.

## Discussion

Children diagnosed with autism are typically portrayed as being significantly different from other children. The experiences of children diagnosed with autism are defined as either positive or negative [42]. Conversely, it has been reported that being diagnosed with ASD in the absence of aftercare avenues, including psychoeducation and assistance in increasing self-understanding, diminishes the benefit of a diagnosis [13, 17]. A diagnosis should deepen one’s understanding of ASD characteristics, and psychoeducation for ASD is crucial to continued understanding and awareness. This must follow a diagnosis to prevent the amplification of related psychological problems such as the perception of self-stigma and lowered self-esteem.

We expect that participants and parents assigned to the ACAT group who experience ACAT will gain a significantly greater understanding of ASD compared to those of the TAU group. Along with an enhanced comprehension of ASD, participants in the ACAT group are also expected to display improved social adaptation, decreased perception of self-stigma, and a reduction in depressive symptoms. Furthermore, the parents of participants are expected to experience less self-stigma, improved mental health, and reinforcement of nurturing resilience. A better understanding of a participant’s ASD characteristics may lead to improved mental health in parents and encourage adaptive behaviors in the participants. Importantly, Gordon et al. [17] reported no significant changes in participants’ self-esteem as a result of an enhanced understanding of ASD. This study assumes that improved self-esteem is experienced after increased social adaptation. However, there was no significant improvement in self-esteem in their study. ACAT may help to increase social adaptation through psychoeducation combined with CBT. We hope that the results of this study will support the benefits of a HF-ASD diagnosis in Japan. Some limitations should be noted, however. First, the comparative effectiveness of the psychoeducation phases of the PEGASUS and ACAT programs is unclear because of the differences in their components. This study is not designed to compare the psychoeducation phases of PEGASUS and ACAT. Hence, we cannot disentangle the specific effects of the psychoeducation phase used in the ACAT program. Second, ACAT is designed as family CBT, and our study design does not include individual CBT as a control group. Individual CBT dealing with problems related to autistic traits may be as effective as ACAT; nevertheless, it is expected that ACAT will be more acceptable and effective for younger participants. Further studies are warranted to establish the effectiveness of these treatment methods.

In conclusion, we expect that participants in the ACAT group will experience significantly increased self-awareness and understanding of ASD symptoms compared to those in the TAU group. We also expect the ACAT group to experience significantly improved social adaptation and mental health if the children and parents in this group gain an adaptive comprehension of their own autistic traits during the sessions. This intervention may contribute to the establishment of an effective treatment strategy based on the evidence gleaned from adolescents with ASD.

## Trial status

The protocol version number

May 30, 2017, version 1.0 (first edition); August 20, 2017, 2.0 edition; September 25, 2017, 3.0 edition; February 19, 2018, 3.5 edition; March 18, 2018, 4.0 edition; April 18, 2019, 4.5 edition; September 5, 2019, 5 edition

The date recruitment began: January 06, 2018

The approximate date when recruitment will be completed: May 2020.

## Data Availability

The datasets generated and analyzed during the current study are not publicly available because analysis during research is prohibited; however, they can be made available upon reasonable request through the corresponding author.

## Abbreviations

ACAT: Aware and Care for my Autistic Traits
ADOS-2: Autism Diagnostic Observation Schedule Second Edition
ASD: Autism spectrum disorder
ADI-R: Autism Diagnostic Interview-Revised
ADHD-RS: ADHD Rating Scale-IV
AKQ: Autism Knowledge Quiz
AKQ-C: Autism Knowledge Quiz-Child
BACE-3: Barriers to Access to Care Evaluation scale version 3
CBT: Cognitive-behavioral therapy
COMB: Combination treatment group
CONSORT: Consolidated Standards of Reporting Trials
DSRS-C: Depression Self-Rating Scale for Children
EEG: Electroencephalogram
GHQ-12: The 12-item General Health Questionnaire
HF-ASD: High-functioning ASD
IQ: Intelligence quotient
M.I.N.I.: Mini-International Neuropsychiatric Interview
PACT: Parent-mediated communication-focused treatment in children with autism
PEGASUS: PsychoEducational Groups for Autism Spectrum Understanding and Support
PREQ: Parenting Resilience Elements Questionnaire
SDQ: Strengths and Difficulties Questionnaire
SPIRIT: Standard and Protocol Items: Recommendations for Interventional Trials
TAU: Treatment-as-usual
Vineland-2: Vineland Adaptive Behavior Scales second edition
WAIS-III: Wechsler Adult Intelligence Scale-III
WISC-IV: Wechsler Intelligence Scale for Children, Fourth Edition
